# *Cannabis*-derived cannabidiol and nanoselenium improve gut barrier function and affect bacterial enzyme activity in chickens subjected to *C. perfringens* challenge

**DOI:** 10.1186/s13567-020-00863-0

**Published:** 2020-11-23

**Authors:** Paweł Konieczka, Dominika Szkopek, Misza Kinsner, Bartosz Fotschki, Jerzy Juśkiewicz, Joanna Banach

**Affiliations:** 1 Department of Animal Nutrition, The Kielanowski Institute of Animal Physiology and Nutrition, Polish Academy of Sciences, Instytucka 3, 05-110 Jabłonna, Poland; 2 Department of Poultry Science, University of Warmia and Mazury, Oczapowskiego 5, 10-718 Olsztyn, Poland; 3 Institute of Animal Reproduction and Food Research, Polish Academy of Sciences, Tuwima 10, 10-748 Olsztyn, Poland; 4 Institute of Natural Fibres and Medicinal Plants, Wojska Polskiego 71b, 60-630 Poznań, Poland

**Keywords:** cannabidiol, selenium nanoparticles, necrotic enteritis, gut health, microbiota activity, broiler chicken

## Abstract

Revealing the multifocal mechanisms affecting cross-talk between *Clostridium perfringens* pathogenesis and the host response is an urgent need in the poultry industry. Herein, the activity of *Cannabis sativa*-derived cannabidiol (CBD) and selenium nanoparticles (Nano-Se) in modulating the host response to *Clostridium perfringens* challenge was investigated in broiler chickens subjected to a mild infection model. The infected chickens exhibited no clinical manifestations, confirming the potential hazard of pathogen transmission to the food chain in the commercial sector. However, both CBD and Nano-Se affected the responses of chickens to *C. perfringens* challenge. The beneficial actions of both agents were manifested in the upregulated expression of genes determining gut barrier function. Both CBD and Nano-Se promoted shifts in gut bacterial enzyme activity to increased energy uptake in challenged chickens and upregulated potential collagenase activity. There was no opposite effect of CBD and Nano-Se in mediating the host response to challenge, whereas an additive effect was evidenced on the upregulation of gene determining gut integrity. Collectively, these findings indicate that understanding the action mechanisms of CBD and Nano-Se is of great interest for developing a preventive strategy for *C. perfringens* infection in broilers.
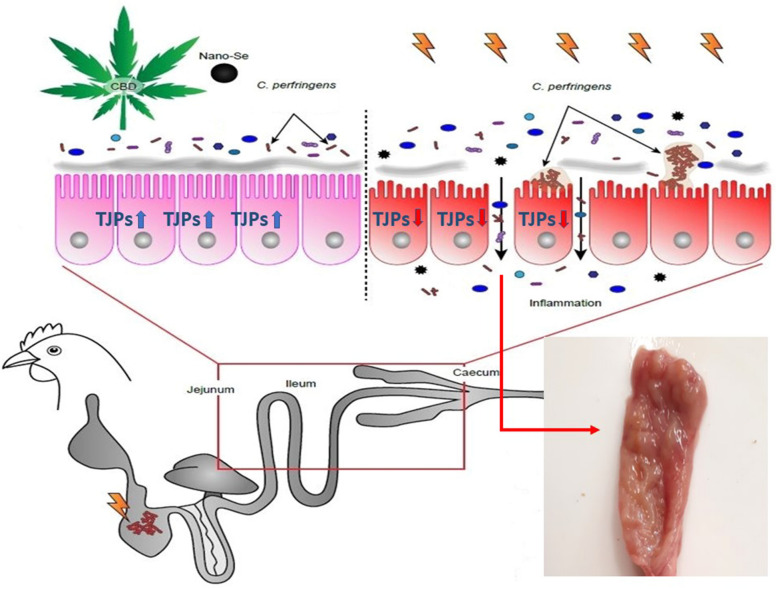

## Introduction

Poultry production is steadily increasing worldwide. In addition to certain welfare issues associated with intensive rearing systems, there are new challenges that not only are potentially hazardous for birds but also could lead to contamination of final products for human consumption. Necrotic enteritis (NE) caused by the anaerobic bacterium *Clostridium perfringens* (or *C. perfringens*) is responsible for major economic loss to the poultry industry due to compromised bird performance and increased rates of morbidity and mortality [1]. This issue has attracted great attention since the preventive use of antibiotics in farm animals has been banned [2]. Thus, there is an urgent need in the poultry sector to develop an effective strategy based on dietary intervention that can limit or prevent the occurrence of NE. Numerous strategies have been investigated to prevent or reduce the negative impact of *C. perfringens* in birds, but the obtained data are inconclusive, mainly because the pathogenic mechanisms of this pathogen are poorly understood [3–6]. Two main factors are responsible for the inconsistent outcomes among studies: (i) the effects of NE-predisposing factors, including coccidia coinfection, nutrition, and bird age [7], and (ii) the basing of conclusions on results obtained/extrapolated from clinical cases with differences in NE disease severity (i.e., subclinical vs. severe). For example, the magnitude of NE lesions affects the expression of genes associated with mucin synthesis in gut tissue [8]. This was evidenced in a study by Forder et al. [9], who found a depressed mRNA level of MUC2 and MUC13 gene expression in the gut tissue as a result of a greater severity of NE in broilers. The authors speculated that it might be associated with an increased chance of further infection. However, considering the current implications for the poultry sector, a focus on understanding the mechanism underlying mild NE is very important, particularly in broiler chickens, because broilers are raised under commercial conditions for an extremely short duration (6–7 weeks). Thus, potential interventions to help broilers recover from advanced NE during the final growth period are rather scarce. In contrast, birds with mild NE typically do not exhibit any symptoms or disorders; thus, these birds might serve as a vector for the delivery of *C. perfringens* and/or its toxins to the food chain [10, 11].

The major site of *Clostridium perfringens-*host interactions in chickens is the gastrointestinal tract (GIT). Thus, this environment is an important anatomical region in which to investigate mechanisms involved in the response to *Clostridium perfringens* challenge and for the development of agents that may potentially regulate this response [12]. Identifying the mechanisms by which NE can be limited may provide new insights into biosecurity. Bioactive components with potential modulatory activity towards GIT health include cannabinoids (CBD) from cannabis plants (*Cannabis sativa*), which have recently attracted increasing attention [13]. Research on the therapeutic potential of CBD has led to many important discoveries in medicine, including the discovery of the action of CBD in regulating food intake, nausea, emesis, gastric secretion, gastroprotection, GIT motility, ion transport, visceral sensation, intestinal inflammation and cell proliferation in the gut [13, 14]. Thus, potential regulation by CBD may be an effective intervention in the management of NE at the initial stage. Although the anti-inflammatory activity of CBD has been extensively studied in human medicine, its influence on GIT function, including immune competence, in veterinary practice is unknown because of the difficulty in identifying the precise dose of CBD for animal treatment. Another agent that exhibits biological activity in the GIT is selenium (Se). Accumulating evidence has shown that the Se status in birds directly affects the immune system, including aspects such as disease resistance, survival, growth performance, fertility, and metabolic processes that determine meat quality [15–18]. However, a recent report indicated that Se has indirect regulatory actions on the GIT through modulation of a host protective response to NE challenge and can alter the residual microbiota, contributing to improved intestinal physiology, allowing increased nutrient absorption and enhancing the immune response to NE [19]. Because nanosized selenium particles (Nano-Se) have been reported to have higher bioavailability but lower toxicity than Se in other forms [20], they could be used to effectively modulate GIT functionality while maintaining compliance with nutritional safety recommendations. Therefore, in this study, we applied—for the first time in a chicken model—dietary treatment with CBD extracted from hemp and with Nano-Se to investigate the activity of these agents in modulating the gut response to *Clostridium perfringens* infection. Additionally, we adopted a challenge model that was effective in the development of mild lesions in broilers. This model was meant to be helpful for investigating changes in bird responses occurring in non-advanced NE and will likely have potential applications in NE-preventive studies and implications for the poultry industry.

## Materials and methods

### Chemical composition of cannabis extract and Nano-Se

Hemp panicles (*Cannabis sativa*) obtained from plants were harvested in 2019 at the Institute of Natural Fibres & Medicinal Plants, Poznań, Poland. Plant material was collected, cut and dried at room temperature. The supercritical carbon dioxide extract of hemp was obtained from the Supercritical Extraction Plant, Institute of New Chemical Synthesis, Puławy, Poland. Parameters of extraction: pressure; 250 bar, temperature; 60 °C, and flow rate of 40 kg CO_2_/1 kg of spent hemp. Hemp extract contained 12% CBD, 0.49% tetrahydrocannabinol and 0.38% tetrahydrocannabinolic acid (Institute of Natural Fibers and Medicinal Plants, Poznań, Poland). Nano-Se was used in the form of a nanopowder with an average particle size of 10–45 nm, specific surface area (SSA) of approximately 30–50 m^2^/g, and purity of 99.9%, according to the manufacturer’s declaration (American Elements, CA, USA).

### Bacterial strain and culture conditions

*C. perfringens* type G strain 56 was isolated from cases of infection in Belgium. The strain was previously confirmed to be α-toxin- and NetB toxin-positive and β-toxin- and enterotoxin-negative according to supplier declaration (Ghent University, Merelbeke, Belgium). The strain was grown anaerobically in autoclaved brain heart infusion broth (Sigma-Aldrich) containing beef heart (5 g/L), calf brain (12.5 g/L), disodium hydrogen phosphate (2.5 g/L), D(+)-glucose (2 g/L), peptone (10 g/L), and sodium chloride (5 g/L) at 37 °C overnight in a jar containing an atmosphere of 10% CO_2_, 10% H_2_, and 80% N_2_.

### Chicken experiment and diets

A total of 360 one-day-old male broilers of the Ross 308 strain purchased from a local commercial hatchery were randomly distributed among the five dietary treatment groups with 8 replicate cages of nine chickens per group. Chickens were fed a standard starter diet for Ross 308 broilers for 8 days. The conditions of the room were maintained according to standard management practices for commercial chicken houses. Beginning on day 9, chicks were transitioned to a grower diet, which was formulated to meet or exceed the requirements for broiler chickens [21]. The basal grower diet based on wheat (50.76%), soybean meal (21.76%), triticale (15.54%), fish meal (5.18%), and rapeseed meal (4.15%) made the gut environment favourable for *C. perfringens* growth. The chickens were fed a control grower diet (unchallenged negative CON group and challenged positive CON group), CON supplemented (on top) with 15 g/kg *Cannabis sativa* extract (CBD treatment group), a diet supplemented with Nano-Se instead of inorganic Se (Nano-Se treatment group), or a diet supplemented with both additives (CBD + Nano-Se treatment group). All grower diets were cold-pelleted in our laboratory in a CL-2 CPM (CA, USA) laboratory pellet mill.

### Establishment of the experimental NE model

Infection of chickens was performed according to a previously described protocol with some modifications [22]. At 15, 16, 17 and 18 days of age, chickens were infected with 1 mL (*per os* directly into the crop) of overnight culture *Clostridium perfringens* inoculate (as described above), prepared fresh for each day of challenge, whereas chickens in the nonchallenged group received the same dose of sterilized broth medium. Before *C. perfringens* challenge, 1 mL of a coccidial cocktail containing the following *Eimeria* (*E*) species was administered to all birds at 14 and 15 days of age to create a favourable gut environment for *C. perfringens* colonization: *E. acervulina*, 5000 oocytes; *E. maxima*, 3500 oocytes; *E. mitis*, 5000 oocytes; *E. praecox*, 5000 oocytes; *E. tenella*, 5000 oocytes (Laboratorios HIPRA S.A., Spain).

### Evaluation of chicken performance

The body weight (BW) and feed intake (Fint) of the chickens were recorded, and performance indices, including the FCR, were calculated for various periods (challenge period, postchallenge period, and all experimental periods) to investigate the specific response of the chickens to the dietary treatments throughout the experimental duration.

### Determination of the collagen level and collagenase concentration in gut tissue

The collagen level in gut tissue was measured as the hydroxyproline content according to the protocol described by Gawronska-Kozak [23]. In brief, samples of gut tissue (middle jejunum) were collected from 8 chickens in each dietary treatment group at 23 and 35 days of age (n = 8). Mucosa samples were homogenized with a glass homogenizer on ice in 2 mL of cold (4 °C) phosphate-buffered saline (Sigma-Aldrich by Merck) and stored overnight at 4 °C. To analyse the level of total collagen, a total of 0.20–0.25 g of mucosa was used. Subsequently, 0.5 mL aliquots were hydrolysed with 0.25 mL of 6 N HCl for 4.5 h at 120 °C. Hydroxyproline (Sigma-Aldrich by Merck) concentrations from 0 to 20 μg/mL were used to construct a standard curve. Twenty microlitres of each sample and the standard dilutions were added to a well plate and incubated for 20 min at room temperature with 50 μL of chloramine T solution (282 mg of chloramine T, 2 mL of n-propanol, 2 mL of distilled water and 16 mL of citrate acetate buffer [5% citric acid, 7.24% sodium acetate, 3.4% sodium hydroxide and 1.2% glacial acetic acid]). Next, 50 μL of Ehrlich's solution (2.5 g of 4-[dimethyloamino] benzaldehyde, 9.3 mL of n-propanol and 3.9 mL of 70% perchloric acid) was added, and the plate was incubated for 15 min at 65 °C. Samples were then cooled, and the plate was read in a microplate reader (Multiskan Sky Microplate Spectrophotometer, Thermo Fisher Scientific) at 550 nm.

Collagenase concentration was measured in the same tissue samples with the corresponding enzyme-linked immunosorbent assay (ELISA) kits (LS BioScience, Seattle, WA, USA) according to the manufacturer’s protocol. In brief, gut samples were collected and rinsed in PBS to remove digesta. Thereafter, the samples were homogenized in 10 mL of PBS with a glass homogenizer on ice and subsequently lysed by ultrasonication. The homogenate was centrifuged at 5000 × *g* for 5 min and was then stored at − 80 °C for further analysis. The standard curve was generated using known antigen concentrations at a wavelength of 450 nm, and the enzyme concentrations in the samples were determined by comparison to the standard curve. Each sample was measured in duplicate, and a standard curve was plotted separately for each ELISA plate.

### RNA extraction and real-time quantitative PCR

Gut samples collected from 23-day-old chickens were additionally analysed by RT-PCR to measure the mRNA expression levels of GLP2, HSP70, TLR4, JAM2, ZO-1, and TFF2. RNA was isolated from tissue according to the protocol described by Konieczka et al. [24] A GeneMATRIX Universal RNA Purification Kit (EURX Ltd., Gdańsk, Poland) was used to isolate total RNA.

The yield of isolated RNA was estimated spectrophotometrically (Nanodrop, NanoDrop Technologies, Wilmington, DE, USA), and the integrity was assessed electrophoretically by separation on an agarose gel. Subsequently, RNA isolated from gut tissue (800 ng/mL) was reverse transcribed to complementary DNA (cDNA) using an NG dART RT Kit (EURX Ltd., Gdańsk, Poland) according to the manufacturer’s protocols. Primers specific for each gene were designed using Primer 3 software (Whitehead Institute, Cambridge, MA, USA) and synthesized by Genomed (Warsaw, Poland) (Table 1). Real-time qPCR was performed in a Bio-Rad CFX 96 thermocycler (Bio-Rad Laboratories, Inc., CA, USA) according to the following programme: (i) enzyme activation (one cycle at 95 °C for 15 min), (ii) denaturation (35 cycles at 95 °C for 10 s), (iii) annealing (35 cycles at 55–58 °C for 10 s) and (iv) elongation (35 cycles at 72 °C for 20 s). Melt curve analysis was performed from 65–95 °C at 0.1 °C/s intervals. Each reaction included a negative control (without the cDNA template). The samples were analysed in duplicate. Gene expression levels were normalized with the comparative quantitation option of the Rotor qPCR Bio-Rad CFX 96 (Bio-Rad Laboratories, Inc., CA, USA), and the values were calculated using the supplied Expression Software Tool. The glyceraldehyde-3-phosphate dehydrogenase (GAPDH) and β-actin (ACTB) genes were used as endogenous controls for normalization of gene expression.

**Table 1:** Genes and primers used in the study.

Gene	Primer	Sequence (5′-3′)	Melting temperature (°C)	Product size (nt)	GenBank accession no.
ACTB	Forward	CGGACTGTTACCAACACCCA	58	115	NM_205518
	Reverse	TCCTGAGTCAAGCGCCAAAA			
GAPDH	Forward	GCACGCCATCACTATCTT	58	82	NM_204305
	Reverse	GGACTCCACAACATACTCAG			
ZO-1	Forward	TCGCTGGTGGCAATGATGTT	58	89	XM_413773
	Reverse	TTGGTCTCCTTCCTCTAATCCTTCTT			
GLP2	Forward	TGTGTTCAGACGGTAAGG	58	127	NM_001163248
	Reverse	TCATCCAGTGCCATCTTC			
HSP70	Forward	GGCAATAAGCGAGCAGTG	58	146	NM_001006685
	Reverse	CGAGTGATGGAGGTGTAGAA			
TFF2	Forward	ACTACCCTACTGAGAGAACAAA	58	143	XM_416743
	Reverse	CTGAAGAACCTGCTCAACTG			
TLR4	Forward	CAAGCACCAGATAGCAACA	58	146	FJ915527
	Reverse	CACTACACTACTGACAGAACAC			
JAM2	Forward	TCCTCCCACTACTCCAATATG	58	134	XM_026849998
	Reverse	ACTGCCTGTTCCTGTCTT			

*ACTB* β-actin, *GAPDH* glyceraldehyde-3-phosphate dehydrogenase, *ZO-1* Zonula occludens-1, *GLP2* Glucagon-like peptide-2, *HSP70* Heat Shock Protein 70, *TFF2* Trefoil Factor 2, *TLR4* Toll-like Receptor 4, *JAM2* Junctional Adhesion Molecule 2.

### Measurement of extracellular and intracellular activity of glycolytic enzymes

The enzymatic activity of the caecal microbiota as a response to dietary treatments was evaluated based on the extra- and intra-cellular activity of bacterial enzymes. The caecal activity of the enzymes (α- and β-glucosidase, α- and β-galactosidase, β-glucuronidase, α-arabinopyranosidase, and β-xylosidase) was determined based on the amounts of *p*-nitrophenol (PNP) or *o*-nitrophenol (ONP) liberated from the respective nitrophenylglucoside substrates, as described [25] and adopted previously [26]. Enzyme activities were measured in caecal digesta collected from chickens at 23 and 35 days of age. In brief, the substrate solution (0.3 mL; 5 mM) and caecal digesta dilution (0.2 mL; 1:10 v/v; centrifuged at 7211×*g* for 15 min) were prepared in 100 mM phosphate buffer (pH 7.0) and were then mixed and incubated at 39 °C. The activity of enzymes (micromoles per hour per gram of digesta) released from the cells into the caecal environment was measured by quantifying PNP or ONP at wavelengths of 400 and 420 nm, respectively. The reaction was terminated with 0.25 M cold sodium carbonate (1 mL). The procedure described above was used to measure extracellular activity. Additionally, the total activity (extracellular + intracellular) of the respective enzymes was measured. For this measurement, a similarly diluted caecal sample containing microbial cells was disrupted with glass beads and a FastPrep-24 homogenizer (MP Biomedicals, Santa Ana, CA). The rest of the procedure was identical to that used to measure extracellular enzymatic activity. Intracellular activity was calculated by subtracting the extracellular activity from the total activity. Standard curves for PNP and ONP were used in the respective calculation formulas. Extracellular enzymatic activity was also expressed as a percentage of total enzymatic activity.

### Statistical analysis

Data are presented as the means (n = 8 chickens per group), and variability is expressed as the pooled standard error of the mean (SEM) values. Differences between groups were assessed using one-way ANOVA with the least significant difference (LSD) test. The significance level was set at P < 0.05. Correlations between tissue collagen content and collagenase concentration were assessed with Pearson correlation analysis. Statistical calculations were performed in STATGRAPHICS Centurion XVI ver. 16.1.03 software.

## Results

### Response of chickens to *C. perfringens* infection

The observation of the gut structures showed no development of advanced infection (data not shown). Different regions of the GIT, including the duodenum, jejunum, and ileum, exhibited no signs of necrosis, which in advanced stadium might be manifested as confluent mucosal necrosis of large parts of the gut segments, a collapsed intestinal lumen, a lack of turgor, a thin and friable intestinal wall, advanced necrosis of the gut mucosa, and apparent multifocal haemorrhage in different areas depending on advancements [3, 27]. Thus, the infection model applied herein was suitable for the investigation of the host response to *Clostridium perfringens* infection of a low severity.

### Performance response to treatments

**Figure 1:**
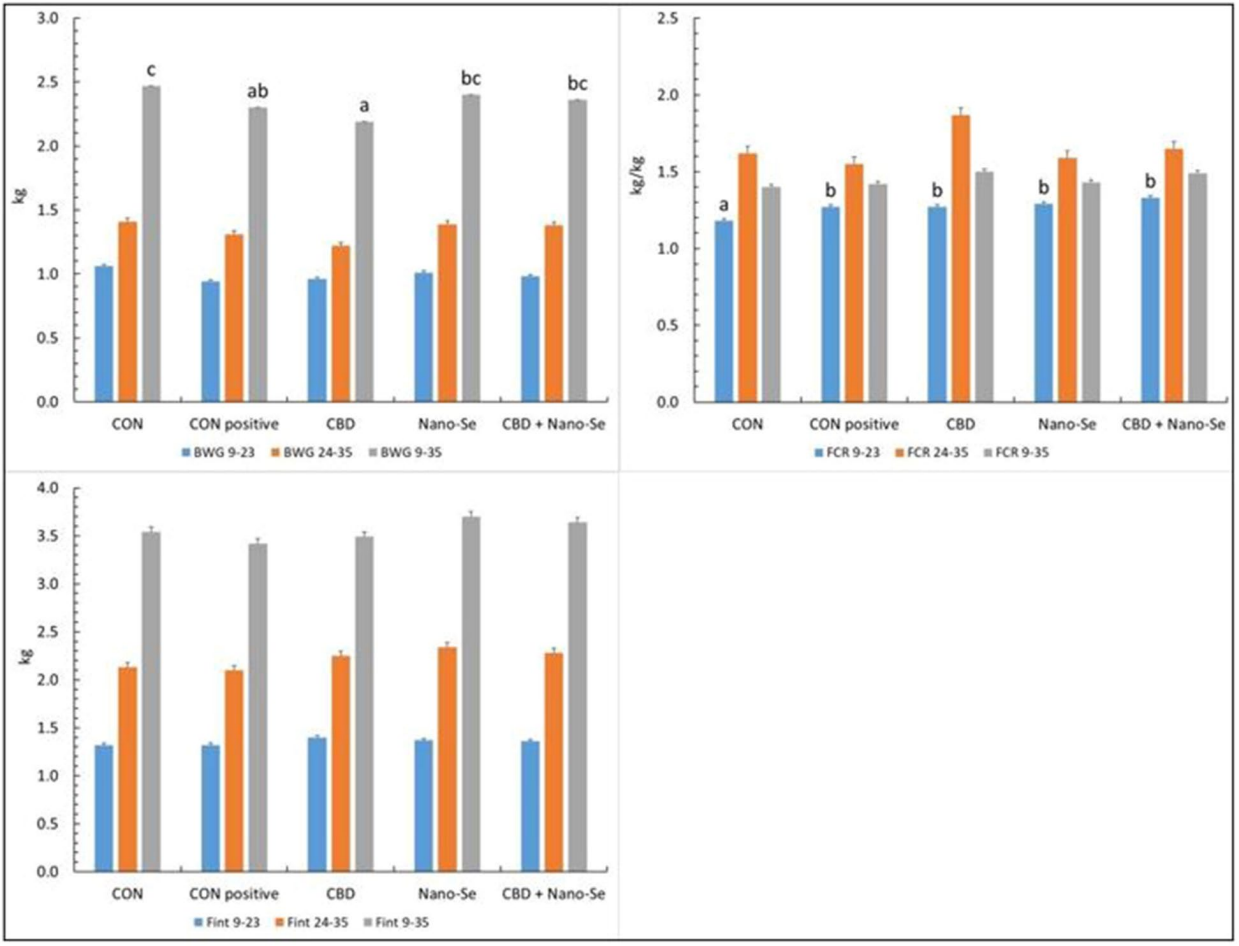
**The body weight gain (BWG), feed intake (Fint), and feed conversion ratio (FCR) were calculated for different experimental periods.** The chickens were fed a control diet (CON), CON supplemented with 15 g of hemp extract (CBD)/kg of diet, CON supplemented with 0.5 mg of nanosized selenium particles (Nano-Se)/kg of diet, or CON supplemented with both additives (CBD + Nano-Se). At 15, 16, 17 and 18 days of age, chickens in each experimental group and the positive control group (CON-positive) were orally infected with 1 mL of 10^8^ CFU/mL *C. perfringens* inoculate. A coccidial cocktail (1 mL) was administered before *C. perfringens* challenge to all chickens. ^a,b,c^The different letters indicate significant differences (P < 0.01). The error bars indicate the mean standard error values for the 8 chickens in each dietary treatment group.

We studied the response of chickens to challenge and to dietary treatments in different stages of growth to gain deeper insight into the response of chickens during and after challenge. The performance response of the chickens to the dietary treatments calculated for different experimental feeding periods is shown in Figure 1. The body weight gain (BWG) in the day 9–23 and day 24–35 feeding periods did not differ between the experimental groups (P > 0.05), whereas the BWG calculated for the overall experimental diet feeding period was significantly lower in the challenged positive CON group and the CBD group than in the unchallenged negative CON group (P < 0.01). The Fint was similar among all treatment groups for all feeding periods (P > 0.05). However, the feed conversion ratio (FCR) was compromised in all experimental groups compared to the unchallenged negative CON group in the day 9–23 feeding period (P < 0.01) but was not affected in the other feeding periods (P > 0.05).

### Regulatory effect of CBD and Nano-Se on collagen degradation and collagenase concentration in gut tissue

**Figure 2:**
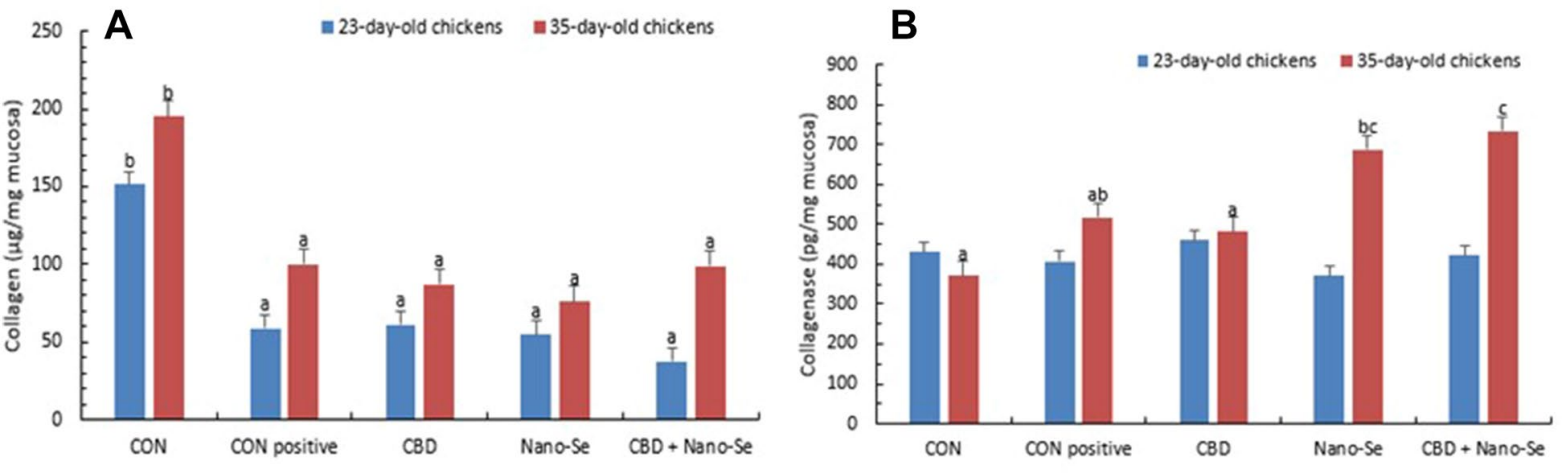
**Collagen content and collagenase concentration in the gut tissue of chickens.** Chickens were fed a control diet (CON), CON supplemented with 15 g of hemp extract (CBD)/kg of diet, CON supplemented with 0.5 mg of nanosized selenium particles (Nano-Se)/kg of diet, or CON supplemented with both additives (CBD + Nano-Se). At 15, 16, 17 and 18 days of age, the chickens in each experimental group and the positive control (CON-positive) group were orally infected with 1 mL of 10^8^ CFU/mL *C. perfringens* inoculate. A coccidial cocktail (1 mL) was administered before *C. perfringens* challenge to all chickens. ^a,b,c^The different letters indicate significant differences (P < 0.01). The error bars indicate the mean standard error values for the 8 chickens in each dietary treatment group.

Challenge with *C. perfringens* resulted in a significantly lower collagen content in gut tissue than that observed in unchallenged chickens (Figure 2). This phenomenon was observed in chickens at 23 days and 35 days of age (P < 0.001); moreover, the dietary treatments did not counteract this effect in chickens of either age (panel A). Regarding the collagenase concentration in gut tissue (panel B), in 23-day-old chickens, no significant differences were observed among the treatments, whereas in the older chickens, feeding with Nano-Se significantly upregulated collagenase concentration compared to that in the unchallenged negative CON group, and feeding with CBD + Nano-Se upregulated collagenase concentration compared to that in both the unchallenged negative CON group and CON-positive groups (P = 0.004). No correlation was observed between collagen content and collagenase concentration in 23-day-old chickens (R = 0.236, P = 0.142), whereas a weak negative correlation between collagen content and collagenase concentration was observed in the gut of older chickens (R =  − 0.344, P =  0.030).

### CBD- and Nano-Se-mediated changes in the transcript levels of select genes determining gut integrity in challenged birds

**Figure 3:**
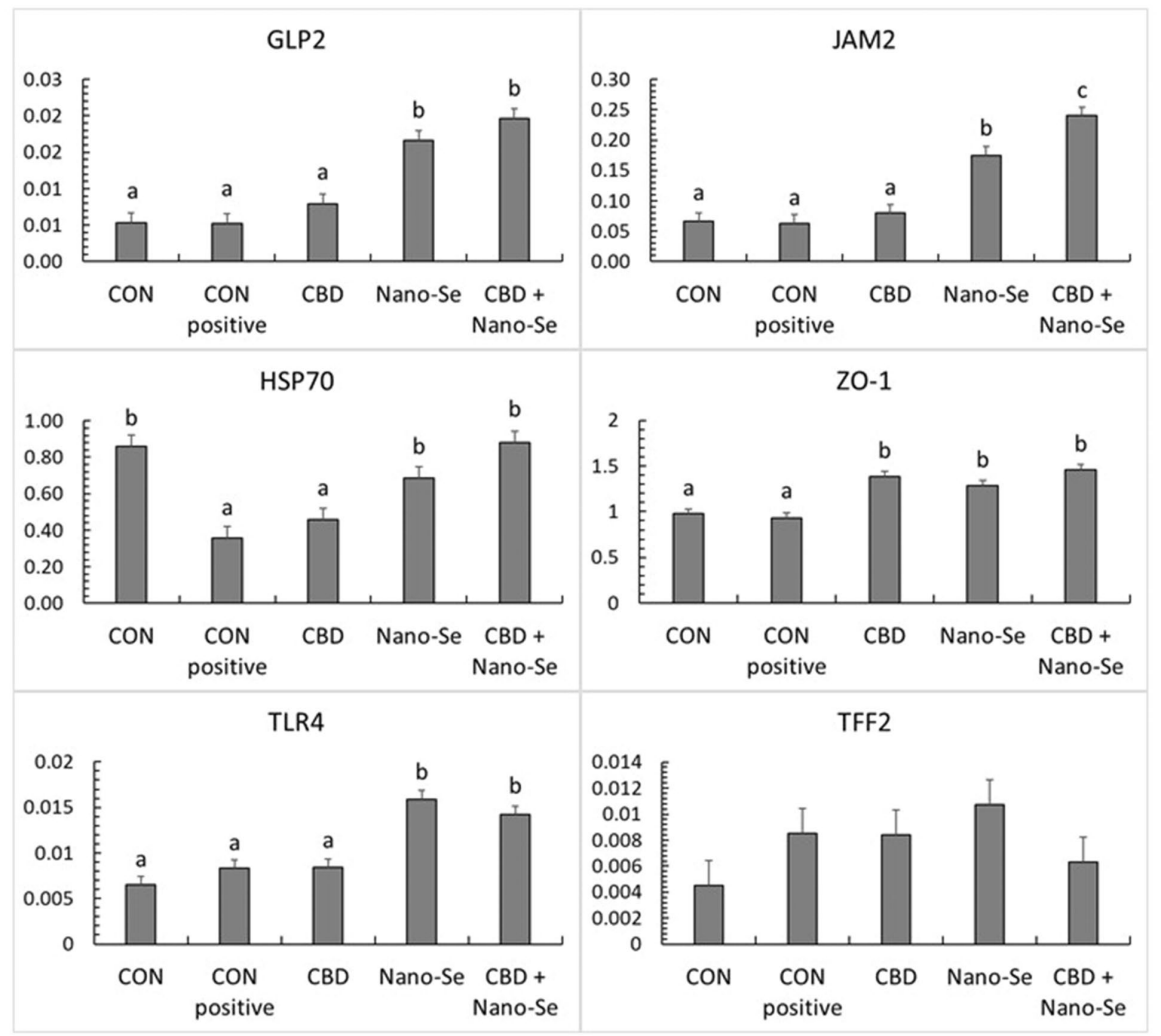
**The relative mRNA expression levels (normalized to the β-actin expression level as the most accurate endogenous control gene) of glucagon-like peptide 2 (GLP2), heat shock protein (HSP70), Toll-like receptor 4 (TLR4), junctional adhesion molecule 2 (JAM2), zonula occludens-1 (ZO-1), and trefoil factor 2 (TFF2) in the gut tissue of chickens.** The chickens were fed a control diet (CON), CON supplemented with 15 g of hemp extract (CBD)/kg of diet, CON supplemented with 0.5 mg of nanosized selenium particles (Nano-Se)/kg of diet, or CON supplemented with both additives (CBD + Nano-Se). At 15, 16, 17 and 18 days of age, the chickens in each experimental group and the positive control (CON-positive) group were orally infected with 1 mL of 10^8^ CFU/mL *C. perfringens* inoculate. A coccidial cocktail (1 mL) was administered before *C. perfringens* challenge to all chickens. ^a,b,c^The different letters indicate significant differences (P < 0.01). The error bars indicate the mean standard error values for the 8 chickens in each dietary treatment group.

The mRNA expression patterns of selected gut integrity-regulating genes in gut tissue are presented in Figure 3. Generally, treatment with Nano-Se and treatment with CBD + Nano-Se significantly upregulated the mRNA expression of genes such as glucagon-like peptide-2 (GLP2), Toll-like receptor 4 (TLR4), and junctional adhesion molecule 2 (JAM2) (all P < 0.001). The expression of the zonula occludens-1 (ZO-1) gene was upregulated in all experimental groups compared to the unchallenged negative CON group and CON-positive groups (P < 0.001). The expression level of the heat shock protein 70 (HSP70) gene did not differ between the challenged positive CON group and CBD treatment groups but was significantly higher in the remaining treatment groups (P = 0.020). The expression level of the trefoil factor 2 (TFF2) gene did not differ among the dietary treatment groups (P > 0.05).

### CBD and Nano-Se affected microbiota enzyme activity

**Figure 4:**
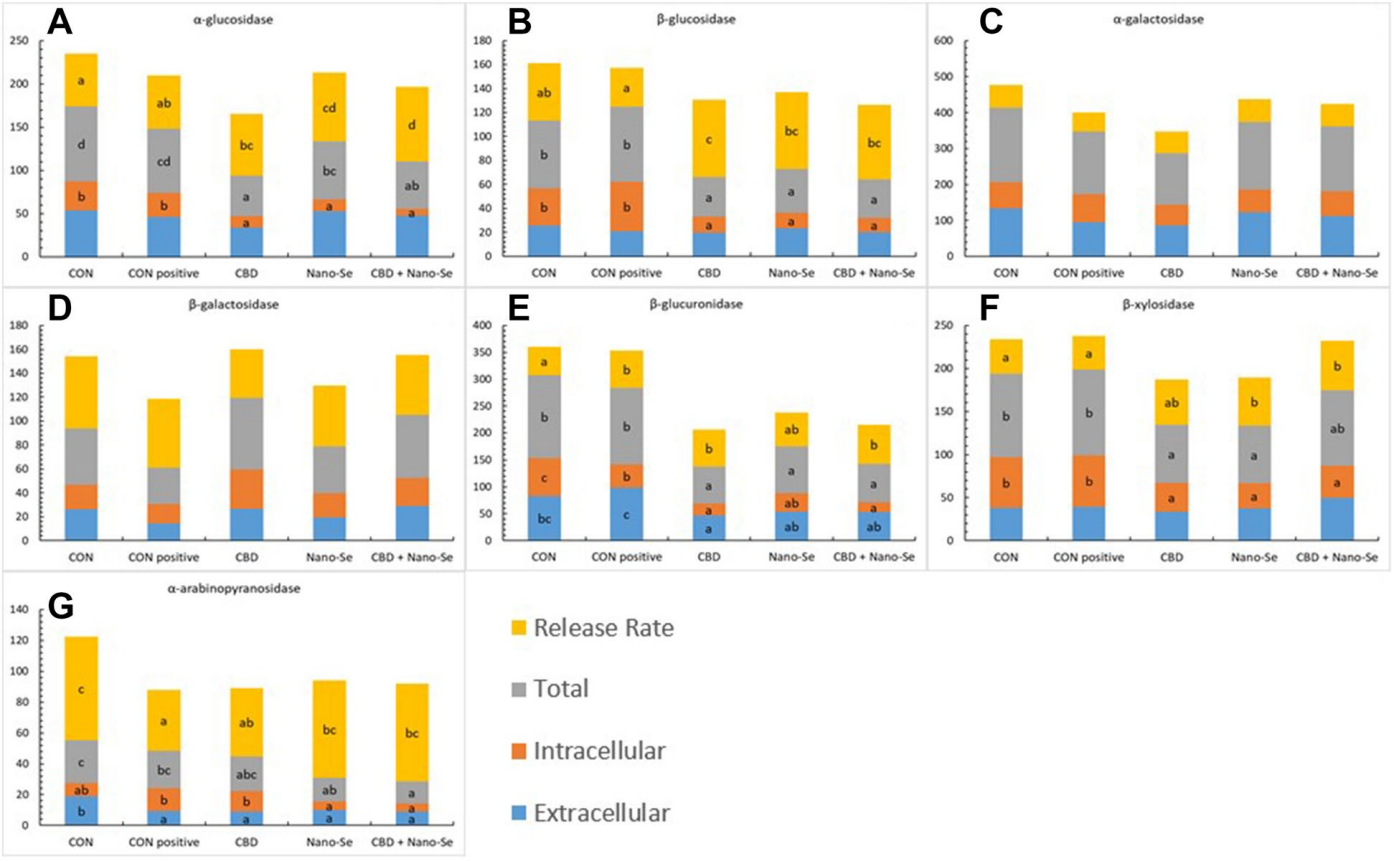
**Bacterial enzyme activity [μmol/(h g protein)] and release rate (%) in the caecal digesta of 23-day-old chickens.** The chickens were fed a control diet (CON), CON supplemented with 15 g of hemp extract (CBD)/kg of diet, CON supplemented with 0.5 mg of nanosized selenium particles (Nano-Se)/kg of diet, or CON supplemented with both additives (CBD + Nano-Se). At 15, 16, 17 and 18 days of age, the chickens in each experimental group and the positive control (CON-positive) group were orally infected with 1 mL of 10^8^ CFU/mL *C. perfringens* inoculate. A coccidial cocktail (1 mL) was administered before *C. perfringens* challenge to all chickens. ^a,b,c^The different letters indicate significant differences (P < 0.01). The error bars indicate the mean standard error values for the 8 chickens in each dietary treatment group.

**Figure 5:**
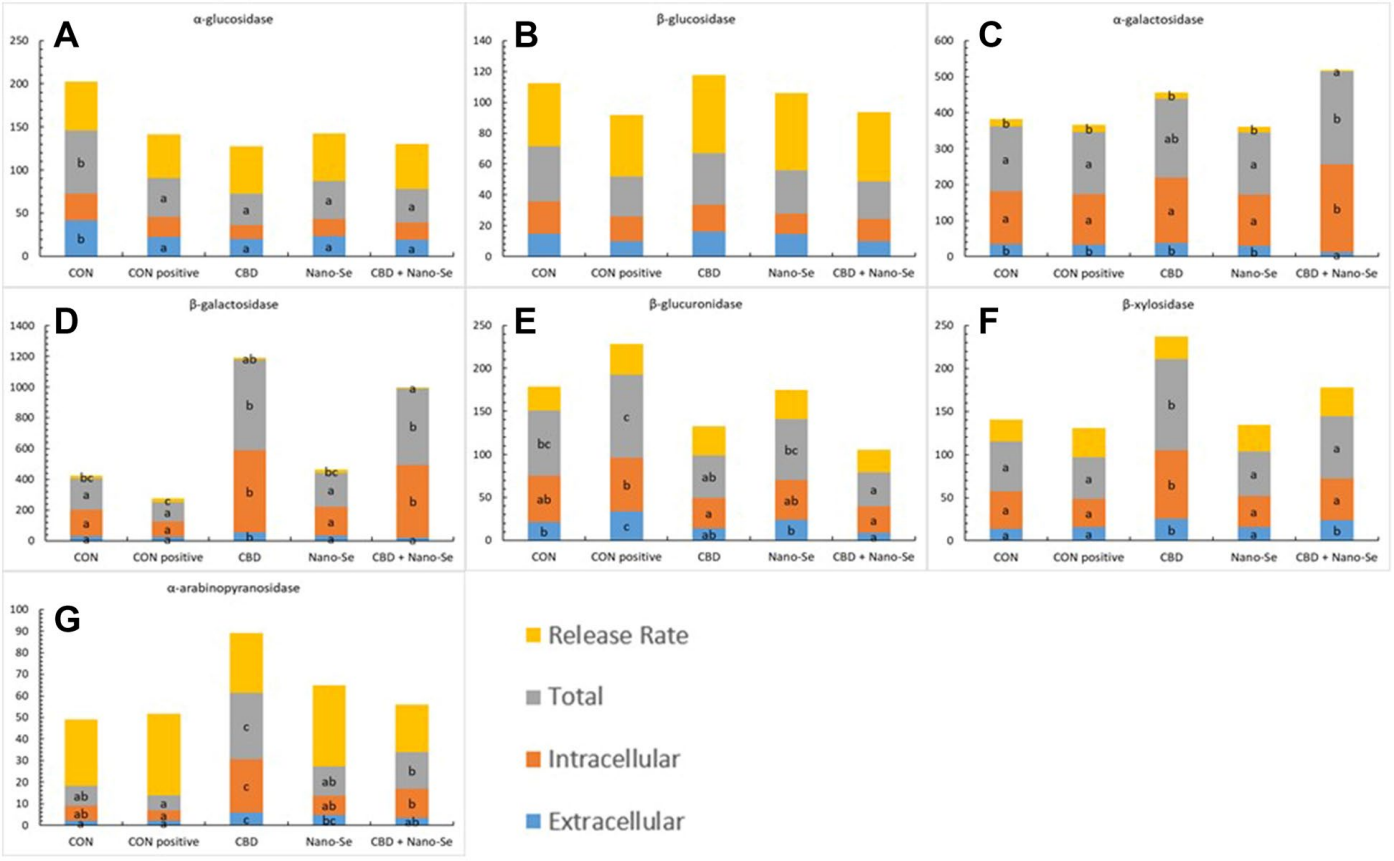
**Bacterial enzyme activity [μmol/(h g protein)] and release rate (%) in the caecal digesta of 35-day-old chickens.** The chickens were fed a control diet (CON), CON supplemented with 15 g of hemp extract (CBD)/kg of diet, a diet supplemented with 0.5 mg of nanosized selenium particles (Nano-Se)/kg of diet, or a diet supplemented with both additives (CBD + Nano-Se). At days 15, 16, 17 and 18 of age, the chickens in each experimental group and the positive control (CON-positive) group were orally infected with 1 mL of 10^8^ CFU/mL *C. perfringens* inoculate. A coccidial cocktail (1 mL) was administered before *C. perfringens* challenge to all chickens. ^a,b,c^The different letters indicate significant differences (P < 0.01). The error bars indicate the mean standard error values for the 8 chickens in each dietary treatment group.

The activity levels of selected bacterial enzymes in the caecal digesta in 23-day-old and 35-day-old chickens are presented in Figures 4 and 5. Both the *C. perfringens* challenge and the dietary treatments caused shifts in the bacterial enzyme activity. In 23-day-old birds, the intracellular activity of β-glucuronidase (panel E) was higher in the unchallenged negative CON group (P < 0.001), but the release rate was lower than that of the CON-positive group (P = 0.039). In the same birds, the extracellular activity and release rate of α-arabinopyranosidase (panel G) were higher in the unchallenged negative CON group than in the CON-positive group (P = 0.016 and P = 0.014, respectively). The remaining enzyme activities did not differ significantly in challenged vs. not challenged birds (P > 0.05). In older birds, the extracellular and total activity of α-glucosidase (panel A) were higher in the unchallenged negative CON group than in the CON-positive group (for both P < 0.001). The extracellular activity of β-glucuronidase (panel E) was higher in the CON-positive group than in unchallenged birds (P < 0.001), whereas other enzyme activities did not differ significantly between challenged and unchallenged birds (P > 0.001). The dietary treatments caused shifts in the intracellular and extracellular activities of different enzymes. In 23-day-old chickens, the intracellular and total activities of α-glucosidase (panel A) were reduced, but the release rate was increased in chickens fed CBD and/or Nano-Se compared chickens fed the control diets (P < 0.001). The same patterns were observed for β-glucosidase activity (panel B), but the release rate was significantly higher in the CBD- and/or Nano-Se-supplemented groups than in the CON-positive group (P < 0.001). The extracellular activity of β-glucuronidase (panel E) was the highest in the unchallenged negative CON group and CON-positive groups (P = 0.004), the intracellular activity was highest in the unchallenged negative CON group (P < 0.001), and the total activity was decreased in all CBD- and/or Nano-Se-supplemented groups (P < 0.001). However, the β-glucuronidase release rate was significantly lower in the unchallenged negative CON group than in the other treatment groups (P = 0.039). The intracellular activity of β-xylosidase (panel F) was significantly lower in all treatment groups than in the unchallenged negative CON group and CON-positive groups (P < 0.001), but the total activity was decreased only in the CBD and Nano-Se treatment groups (P = 0.010). The highest release rates were found in all the CBD and/or Nano-Se supplemented groups, but these rates differed significantly between the Nano-Se and CBD + Nano-Se treatment groups and the unchallenged negative CON group and CON-positive groups (P = 0.033). The extracellular activity of α-arabinopyranosidase (panel G) was highest in the unchallenged negative CON group compared to the other treatment groups (P = 0.016), the intracellular activity was lower in the Nano-Se and CBD + Nano-Se treatment groups than in the challenged positive CON group (P = 0.013), and the total activity was significantly lower in the Nano-Se and CBD + Nano-Se treatments than in the unchallenged negative CON group (P = 0.024). The α-arabinopyranosidase release rate was significantly higher in the unchallenged negative CON group than in the CON-positive and CBD groups (P = 0.014). The activities of these specific enzymes shifted in 35-day-old chickens (Figure 5) as follows: the extracellular and total activities of α-glucosidase (panel A) were reduced in all groups compared to the unchallenged negative CON group (P < 0.001). Among the treatment groups, the extracellular activity of α-galactosidase (panel C) was the lowest, but the intracellular activity was the highest in the CBD + Nano-Se treatment group (P < 0.001). The α-galactosidase release rate was the lowest in the CBD and CBD + Nano-Se treatment groups (P < 0.001). The extracellular activity of β-galactosidase (panel D) was increased significantly in the CBD treatment group (P = 0.002), whereas the intracellular and total activities were increased in the CBD and CBD + Nano-Se treatment groups compared with the remaining treatment groups (P < 0.001). The β-galactosidase release rate was the lowest in the CBD + Nano-Se treatment group (P < 0.001). In the CBD + Nano-Se treatment group, the extracellular and total activities of β-glucuronidase (panel E) were reduced compared with those in the unchallenged negative CON group (P < 0.001 and P = 0.004, respectively). CBD supplementation caused significant increases in the extracellular, intracellular, and total activities of α-arabinopyranosidase (P < 0.001) (panel F) and β-xylosidase (P = 0.013, P = 0.007 and P < 0.001, respectively) (panel G).

## Discussion

To the best of our knowledge, this is the first study investigating the bioactive properties of CBD and Nano-Se in broilers with potential activity towards gut health and functionality modulation. In particular, the mechanism underlying the action of CBD has not yet been fully elucidated, but it may represent a biological activity in the regulation of inflammation by a close affinity to the processes mediating necrosis and inflammation of the chicken gut. Because NE is an urgent problem in the poultry industry, this bioactive agent may represent an interesting solution in preventive strategies. Therefore, additives that can contribute to the health status of birds while maintaining bird performance indices are currently of high interest for the poultry sector.

The performance indices of the chickens reported in the present study confirmed that the challenge only marginally affected their growth. According to other authors [28], subclinical NE was associated with depressed performance and mild lesions in the small intestine. Interestingly, however, during the experimental period in which the challenge was performed (days 9–23), neither the BWG nor Fint of the chickens changed significantly, but the FCR was compromised in all challenged chickens compared to the unchallenged chickens. This pattern suggests that the response of the chickens to infection is more complex than a simple reduction/increase in feed consumption and that mechanisms controlling feed utilization are involved. Calefi et al. [29] demonstrated that gut-brain axis interactions during NE challenge may play a key role in the bird response. Our findings confirmed that infection with *Clostridium perfringens* may occur with no signs of overt growth suppression and is a potential threat to the poultry industry; therefore, this condition warrants special attention.

The occurrence of NE has been linked to the ability of *C. perfringens* to adhere to the host extracellular matrix structures through the adhesive ability to collagen, which is the main structural component of connective tissue and thus potentially the initial pathogenic target [30, 31]. Collagen disruption was clearly observed in our study; the gut tissue collagen content was significantly decreased in both 23- and 35-day-old birds challenged with *C. perfringens*. Regarding the dietary treatments applied in the present study, neither CBD nor Nano-Se (or the combination) significantly affected the collagen content in gut tissue. On the other hand, there was no significant effect of *C. perfringens* challenge on the gut total concentration of collagenase enzymes (negative CON group vs. CON-positive group) in 23- and 35-day-old birds. These results are in contrast to those reported by Van Damme et al. [32], who reported reduced expression of selected host collagenases in necrotic gut tissue as a result of *C. perfringens* challenge. The contradictory findings may indicate that the severity of the infection may play a role in the specific response of host collagenase enzyme activity since in our study, no advanced necrosis developed. However, the total concentration of collagenase enzymes in the gut tissue in older chickens was significantly increased in the Nano-Se treatment group compared to the negative CON group and was significantly increased in the CBD + Nano-Se treatment group compared to both the negative CON group and the CON-positive group.

Collagenase enzymes are members of the matrix metalloproteinase (MMP) family of protease enzymes, whose primary function is degrading and remodelling the extracellular matrix in gut tissue [33, 34]. The secretion of collagenolytic enzymes and activation of host MMP cascades, which induce collagen degradation and, consequently, gut enteritis, have been reported to be key *C. perfringens* virulence factors in NE [35]. Our findings indicated that CBD and Nano-Se did not manifest an opposite action in mediating host collagenase enzyme secretion in the gut tissue, whereas Nano-Se promoted this process. This may be considered unfavourable for the host gut. Aguirre et al. [36] reported that supplementation of the chicken diet with resin acids had a protective effect on the intestinal barrier integrity under optimal (with no challenge) conditions, which were linked to decreased activities of collagen-degrading enzymes in the gut tissue. Another report [37] indicated that raisin acid supplementation improved broiler performance in both optimal and NE-challenged conditions, but the authors failed to show a clear mode of action for this effect; thus, the significant role of collagen-degrading enzymes in mediating the host response to *C. perfringens* challenge cannot be excluded.

Based on our results, there was mostly no significant response as a result of *C. perfringens* challenge in mRNA expression of selected genes in the jejunal tissue investigated as relevant indicators of gut integrity in chickens. This is in contrast to other reports (reviewed by Awad et al.) [38], in which it was revealed that *C. perfringens* infection causes an overall perturbation in the expression of genes determining gut integrity. However, such an effect of *C. perfringens* is mostly reported in birds with advanced forms of infection, which may partially explain the different responses reported in the present study. Tight junction proteins (TJPs) are the key molecules maintaining epithelial barrier integrity. TJPs enable the passage of ions inside cells but prevent pathogens and their toxins from entering cells, thus acting as gatekeepers [39]. Although data regarding the specific action of CBD in the bird gut are lacking, studies in different models suggest that CBD possibly modulates the gut neuro-immune axis and mediates gut inflammation via complex cross-talk with other immune cell types [40]. Alhamoruni et al. [41] demonstrated an enhancing effect of CBD on the mRNA expression of TJPs, including ZO-1, in an in vitro model.

In the present study, CBD and Nano-Se upregulated the expression of ZO-1 and JAM2, which are among the major molecules controlling cell-to-cell adhesion in the gut, while increased expression of TLR4 indicated activation of macrophages as a response to inflammation and augmentation of the host defence against pathogens [42]. Additionally, Nano-Se and Nano-Se + CBD (but not CBD alone) upregulated the expression of the HSP70 gene in the gut tissue. HSP70 belongs to the group of proteins that mediate the stress response in gut tissue, and these proteins are important for adaptation to environmental changes. HSPs are constitutively present in gut tissue and respond to stress-related stimuli [43]. Thus, considering that the HSP70 expression level in our study was similar in both the challenged positive CON group and CBD group but was significantly higher in the remaining treatments, supplementation with Nano-Se was effective in maintaining stress responsiveness in the gut tissue, whereas additional supplementation with CBD did not compromise this response. The action of Se in modulating the response of the broiler gut to challenge has been studied extensively. Se supplementation enhances the production of key components of innate immunity during the initial response to pathogenic stimuli in birds and therefore improves intestinal barrier function [19, 44]. The maximum recommended dose of Se in the broiler diet has been established to be 0.5 mg/kg of feed [45]. However, recently, we documented that even a dose of 0.7 mg of Se/kg of feed was not effective in downregulating oxidative processes in chicken and rat models [46]. The present report demonstrated that Nano-Se, when added at the recommended maximum dose, might have greater potential than other forms of Se to support gut function in chickens. We also provided the first documentation of the potential interaction effect between CBD and Nano-Se in modulating the host response, indicating that the bioactive properties of both agents do not manifest an opposite effect.

Changes in the abundances and metabolic characteristics of the gut microbiota result from their cross-talk with the physiology and diet of the host, which could ultimately influence the specific response of the host to dietary supplements. In contrast to other reports, we did not measure the specific microbiome composition as a response to the dietary treatments, as this parameter varies greatly depending on the conditions [47, 48]. Instead, we investigated changes in the glycolytic activity of the gut microbiota in situ as a mechanism of adaptation for energy uptake to both the applied challenge and dietary treatments, as it is more useful for the present study than changing the bacterial composition. The extracellular enzymatic activity of the microbiome is influenced by the types and abundances of bacterial species residing in the gut and by the rate of release of enzymes from these bacterial cells into the intestinal environment [26]. Therefore, increases in the extracellular activity compared to the total activity (sum of the extracellular and intracellular activities) of those enzymes could be an important adaptive mechanism of intestinal bacteria, as was shown in a different model [49]. We additionally speculate that it could be a potential biomarker of the effect of the applied treatment on the host response. In line with this, the present report demonstrated changes in the activity of selected bacterial enzymes as a result of *C. perfringens* challenge. Particularly indicative was that in 23-day-old birds, there was a shift from intracellular activity towards an increased realizing rate of β-glucuronidase in the caecal digesta comparing the CON-positive group to the unchallenged CON-negative group. It was reported that the increased activity of β-glucuronidase may be indicative of increased abundance of *Clostridium* populations in the gut, and it is associated with the higher risk of glucuronide hydrolysis in the gut lumen, which generates toxic and carcinogenic substances from nontoxic glycosides [50]. In general, the present study demonstrated that the intracellular activity of 5 of the 7 investigated bacterial enzymes was shifted towards an increased rate of release of glycolytic enzymes. More specifically, the greatest shifts were observed in chickens treated with CBD, Nano-Se, and CBD + Nano-Se. Because bacterial enzyme activity depends on the bacterial composition in the digesta, it cannot be excluded that challenging birds with *C. perfringens* altered the microbial balance in the gut; however, the bacterial composition was not analysed in this study. However, a clear response was that treatment with CBD and Nano-Se affected bacterial activity by increasing the enzyme release rate. The increased rate of enzyme release is an adaptive mechanism by which bacteria obtain additional energy from the gut during excessive *C. perfringens* proliferation (the period of challenge). The enzyme release rate could be mediated in both a direct manner (through the ability of CBD and Nano-Se to enter bacterial cells) and an indirect manner (through the induced secretion of gut molecules, i.e., mucins, which are metabolized by bacteria) [51]. This hypothesis is supported by the results of other studies that reported the gut microbiome-modulating properties of CBD and Nano-Se [52–55]. Gut microbes metabolize Se for their own needs to synthesize microbial selenoproteins [56]; however, it is not clear how they interact with CBD. Nevertheless, our report demonstrated that CBD induced a shift in the release of bacterial enzymes in the gut, and this action was supported by Nano-Se. Interestingly, our findings indicate that the microbiome did change the enzymatic activity in the post-challenge period because in older birds, the relationship between intra- and extracellular enzymatic activity was markedly changed. An overall shift in extracellular activity but not an increased enzyme release rate was observed in the younger birds. This pattern might be considered beneficial because it indicates that neither CBD nor Nano-Se increases the energy uptake of gut bacteria under optimal conditions (after recovery from challenge), which could be costly regarding energy demands to the host.

In conclusion, this study provided evidence that broilers may be infected with *C. perfringens* with no clinical manifestations, a condition that is potentially hazardous due to possible pathogen transfer to the food chain. Herein, we showed—for the first time in a chicken model—that CBD and Nano-Se may be able to modulate the response of chickens to *C. perfringens infection*, which may allow time for effective intervention. The beneficial effect of both agents on host physiology was manifested in the support of gut barrier function through increased expression of genes controlling gut integrity. CBD and Nano-Se promoted shifts in bacterial enzyme extracellular activity to increased energy uptake in challenged chickens, and these two agents did not exhibit an opposite effect on mediating the host response to infection. Collectively, these findings indicate that understanding the action mechanisms of CBD and Nano-Se is of great interest for the development of a preventive strategy for NE in broilers.

## Data Availability

The datasets used and/or analysed during the current study are available from the corresponding author on reasonable request.

## Abbreviations

BW: body weight
BWG: body weight gain
CBD: cannabidiol
CON: unchallenged negative control group
FCR: feed conversion ratio
Fint: feed intake
GIT: gastrointestinal tract
GLP2: glucagon-like peptide 2
HSP70: heat shock protein
JAM2: junctional adhesion molecule-2
MMP: matrix metalloproteinase
Nano-Se: selenium nanoparticles
NE: necrotic enteritis
TJPs: tight junction proteins
TLR4: toll-like receptor 4
TFF2: trefoil factor 2
ZO-1: zonula occludens 1
